# Complete Genome Sequence of Allorhizobium vitis Strain K306, the Causal Agent of Grapevine Crown Gall

**DOI:** 10.1128/MRA.00565-20

**Published:** 2020-07-16

**Authors:** Hangwei Xi, Maarten Ryder, Iain R. Searle

**Affiliations:** a School of Biological Sciences, The University of Adelaide, Adelaide, Australia; b School of Agriculture, Food and Wine, The University of Adelaide, Adelaide, Australia; University of Maryland School of Medicine

## Abstract

Here, we present the annotated complete genome sequence of Allorhizobium vitis K306, a phytopathogenic strain causing crown gall of grapevine. The *A. vitis* K306 genome is 5.79 Mb long with 5,199 predicted protein-coding genes and contains 2 circular chromosomes of 3.8 Mb and 1.1 Mb and 2 plasmids, namely, pTiK306 and pTrK306, that are 262 kb and 581 kb, respectively.

## ANNOUNCEMENT

Crown gall is a chronic untreatable disease that occurs on a large number of agronomically important crops worldwide, including grapevine. The causal agent of crown gall disease on grapevine is the bacterium Agrobacterium vitis; however, the bacterium has been reclassified to the genus *Allorhizobium* based on whole-genome phylogeny (1, 2). Virulent *A. vitis* strains harbor a tumor-inducing (Ti) plasmid that, upon infection and transfer of transfer DNA (T-DNA) to the plant cell, encodes enzymes involved in auxin and cytokinin metabolism, causing plant cell enlargement and division, leading to the appearance of tumors. Other T-DNA genes code for the synthesis of low-molecular-weight compounds, known as opines, that are utilized by the inciting bacteria but cannot be metabolized by the host plant (3). The *A. vitis* strain known as K306 is of the OL type, i.e., catabolizes octopine and has a large TA (T-DNA) region (4), and strains of this type catabolize octopine as a sole carbon and nitrogen source (5, 6). To date, only two *A. vitis* genome sequences are available, namely, a complete genome sequence of strain S4 (7) and a draft genome sequence of the type strain K309 (NCPPB 3554) (8).

*A. vitis* K306 was first isolated from a grapevine near Barmera, South Australia, in 1976 (9). A single bacterial colony of K306 from the Kerr collection (University of Adelaide, Waite campus) was inoculated into 5 ml LB broth and grown overnight at 28°C. After the cells were centrifuged and the supernatant discarded, genomic DNA was purified from the cells by using a DNeasy PowerSoil kit (Qiagen). The purified DNA was sheared to 10 to 15 kb utilizing needle shearing, the 10- to 25-kb fragments were size selected by using a Blue Pippin instrument (Sage Science), and then a SMRTbell library was constructed by using the SMRTbell template prep kit 1.0 (PacBio, Menlo Park, CA). Sequencing was performed on a PacBio Sequel instrument that generated 16,604 circular consensus (CCS) reads, with a CCS *N*_50_ value of 8.1 kb and a total of 1.6 Gb of sequence data, at about 250× coverage. The CCS parameters were --minLength=10, --52 maxLength=21000, --minPasses=3, --minIdentity=0.82, --minSnr=2.5, --53 maxPoaCoverage=0, --window=500, --window-min-len=100, --polish, --draft-54 mode=”windowed,” --max-acc-abs-dev=0.05, --max-acc-frac-dev=0.05, --minPredictedAccuracy=0.9, --minReadScore=0.75, --maxDropFraction=0.34, and --56 minZScore=-3.14. The processed reads were assembled by using Flye v2.7 (10).

After assembly by Flye, four contigs were produced that had a combined length of 5.79 Mb and a GC content of 57.5% (Table 1). The K306 genome size and GC content are similar to those of the draft genome of *A. vitis* K309 and the completely assembled *A. vitis* S4 genome (7, 8). The *A. vitis* S4 genome has two circular chromosomes and five plasmids, including the tumor-inducing plasmid pTiS4. After using Geneious Prime 2020.1.1 plugin LASTZ v7.02 (11) to align the four *A. vitis* K306 contigs to the Agrobacterium tumefaciens S4 genome, contig 1 was found to be most similar to chromosome 1, contig 2 was most similar to chromosome 2, and contig 3 was similar to the tumor-inducing pTiS4 plasmid. Accordingly, contig 1 was named chromosome 1, contig 2 was named chromosome 2, and contig 3 was named pTiK306, indicating that it is likely the tumor-inducing plasmid. The latter designation of contig 3 as pTiK306 was confirmed by the presence of a virulence (*vir*) region, genes homologous to known T-DNA genes involved in plant hormone metabolism, and genes related to opine synthesis and catabolism, as expected of a Ti plasmid. We demonstrated that K306 chromosomes 1 and 2, contig 4, and pTiK306 were circular by manually editing the sequence file to add 40 kb of the sequence from the 3′ end to the 5′ end of the contig and identifying spanning sequence reads by using minimap2 v2.0 in Geneious (12). On contig 4 we identified a 120-kb region from 219.3 to 345.3 kb that has a 92.9% average nucleotide identity (ANI) to that of *A. vitis* S4 plasmid pAtS4e (GenBank accession number NC_011981.1). Based on the presence of candidate tartrate dehydrogenases, ttuC_1 and 2, and genes on this contig and the sequence being circular, we name this plasmid pTrK306.

**TABLE 1:** Features of the complete *A. vitis* K306 genome

Feature	Data for:			
	Chromosome 1	Chromosome 2	pTiK306	pTrK306
Size (bp)	3,804,261	1,140,656	262,861	580,927
GC content (%)	57.7	57.6	56.3	56.9
No. of CDS^*a*^	3,474	971	239	515
No. of rRNAs	9	3	0	0
No. of tRNAs	53	4	0	0

a CDS, coding DNA sequences.

The DDBJ Fast Annotation and Submission Tool (DFAST) (13) was used to predict coding genes in the K306 genome. DFAST identified 5,199 protein-coding sequences, 12 rRNAs, and 57 tRNAs (Table 1). Similar to the *A. vitis* S4 genome, no rRNAs or tRNAs were predicted on pTiK306 or pTrK306. Tumor-inducing plasmids have one or more T-DNA regions. On pTiK306, we identified two possible T-DNA regions flanked by left border (LB) and right border (RB) sequences. This is similar to *A. vitis* S4, which has three T-DNA regions (14). The first T-DNA region spans from nucleotide (nt) positions 213415 (LB) to 228573 (RB), and interestingly, adjacent to the LB sequence is a candidate agrocinopine synthase (*acsx*) gene. To further support the hypothesis that K306 produces agrocinopines, we identified three agrocinopine catabolic genes, namely, *accG*, *accF_1*, and *accF_2*, and the regulatory gene *accR*. Agrocinopines have not been described in grapevine galls to date, and hence, the production of agrocinopines, or a related as-yet-undescribed opine, warrants further investigation.

### Data availability.

The complete genome sequence and associated data for *A. vitis* K306 were deposited under GenBank accession number JABFNP000000000, BioProject accession number PRJNA630500, SRA accession number SRR11782079, and BioSample accession number SAMN14825643.
